# Evaluation of the feasibility, safety, and preliminary effectiveness of coil and foam embolization in patients with venous-origin chronic pelvic pain

**DOI:** 10.3389/fmed.2026.1741818

**Published:** 2026-01-22

**Authors:** Yangyang Wang, Fangting Li, Guangying Niu, Wenzhe Zhang, Lin Lu, Kai Zhang, Yongyuan Zhang, Yu Zhang, Xing Chen, Xin Zhao

**Affiliations:** 1 Department of Medical Imaging, The Third Affiliated Hospital of Zhengzhou University, Zhengzhou, China; 2 Department of Medical Imaging, Zhengzhou People’s Hospital, Zhengzhou, China; 3 Department of Gynecology, The Third Affiliated Hospital of Zhengzhou University, Zhengzhou, China

**Keywords:** embolization, ovarian vein, pelvic vein disease, polidocanol sclerotherapy, venous-origin chronic pelvic pain

## Abstract

**Objectives:**

This study aims to evaluate the safety and efficacy of ovarian vein embolization combined with polidocanol sclerotherapy for Venous-origin chronic pelvic pain (VO-CPP) and to elucidate potential risk factors affecting prognosis.

**Methods:**

A total of 158 patients diagnosed with VO-CPP who underwent embolization were enrolled between October 2019 and August 2024. Patients were followed up at 1 month, 3 months, 6 months, and then annually after the embolization through phone calls and outpatient clinic visits. The primary outcome was the proportion of patients achieving an effective response, defined as a decrease in the Visual Analog Scale (VAS) by at least 50% at the final follow-up. Secondary outcomes included technical success, the time to onset of pain relief, complication rates, and risk factors for poor prognosis.

**Results:**

Technical success was achieved in 100% of cases, with most patients experiencing significant pain relief by 3 months post-embolization. The VAS scores at each time point after embolization were significantly lower than those recorded before the procedure, with all differences being statistically significant (*p* < 0.001). The proportions of clinical effectiveness at 6 months post-embolization and at the final follow-up were 83.5 and 75.9%, respectively. Pain-related symptom recurrence occurred in 9.1% (12/132) of patients within 6 to 15 months. Parity ≥3 (OR, 2.266; 95% CI: 1.034–4.969; *p* = 0.041) and estrogen-dependent disease (OR, 2.586; 95% CI: 1.177–5.680; *p* = 0.018) were the independent risk factor for poor prognosis. The complication rate of combined therapy was 7%, with the most common complication being thrombosis of the pelvic veins.

**Conclusion:**

Ovarian vein embolization combined with polidocanol sclerotherapy is a feasible and safe endovascular procedure for the treatment of VO-CPP. Multiparous and comorbidities were the independent risk factor associated with poor prognosis.

## Introduction

The definition of pelvic vein disease (PeVD) is unclear before 2022, which has previously been referred to as pelvic congestion syndrome, Nutcracker syndrome and May-Thurner syndrome, involves valvular insufficiency, reflux and venous obstruction, and usually occurs in the ovaries and internal iliac veins. The clinical manifestations of PeVD are complex, and its symptoms are often variable, leading clinicians to frequently misdiagnose as pelvic inflammatory disease, fibroids, adenomyosis, or endometriosis, which account for about 10% of all outpatient visits (1).

Venous-origin chronic pelvic pain (VO-CPP) is a clinical manifestation of PeVD, defined as pain symptoms that are believed to originate from pelvic organs and last for more than 6 months, affects up to 26% of women worldwide (2, 3).

The symptoms of VO-CPP are heterogeneous in both type and severity, including menstruation-associated chronic pelvic pain, dyspareunia, bladder symptoms, perineal heaviness, etc. These symptoms tend to worsen with fatigue, particularly during sexual intercourse or after prolonged periods of standing or sitting. Due to its nonspecific symptoms, PeVD is easily overlooked and has a significant negative impact on the quality of life in premenopausal women (4, 5).

In 1993, Edwards et al. (6) first reported the treatment of PeVD through ovarian vein embolization. This minimally invasive procedure is typically performed under local anesthesia, facilitating quicker recovery and shorter hospital stays compared to traditional surgical options such as hysterectomy or vein ligation. Various materials can be utilized for vein embolization, including coils, vascular plugs, foam sclerosant, and ethiodized oil. The embolization methods may involve the use of spring coils or vascular plugs to occlude refluxing vessels, and these can be combined with foam hardeners, tissue glue, iodide, and other agents to enhance the embolization effect. Regarding the target veins, Laborda et al. (7) and De Gregorio et al. (8) recommended bilateral embolization of the ovarian and internal iliac veins, while Liang et al. suggested embolizing only the refluxing veins (9). Numerous studies (9–14) have confirmed that transcatheter embolization has emerged as an effective treatment for PeVD and is recommended with a class IIa, level of evidence B recommendation in current guidelines (15, 16). However, due to individual differences in the symptoms experienced by patients, the effectiveness of embolization varies significantly, with reported success rates ranging from approximately 61–92.3% (9, 12, 17, 18). Symptom recurrence occurs in about 7.4 to 42% of cases, predominantly due to recurrent pelvic pain or pelvic varices (19). Currently, the factors associated with pain recurrence or incomplete symptom improvement remain unclear. Therefore, for consistency in the evaluation, we focused on the subgroup of patients with VO-CPP–category S_2_V_2_ in the Symptoms-Varices-Patophysiology (SVP) Classification as it is reported to be the most common occurrence (4).

The objective of the present study is to assess the safety and efficacy of ovarian vein embolization combined with polidocanol sclerotherapy for VO-CPP patients, as well as to elucidate potential risk factors affecting prognosis.

## Materials and methods

**Figure 1:**
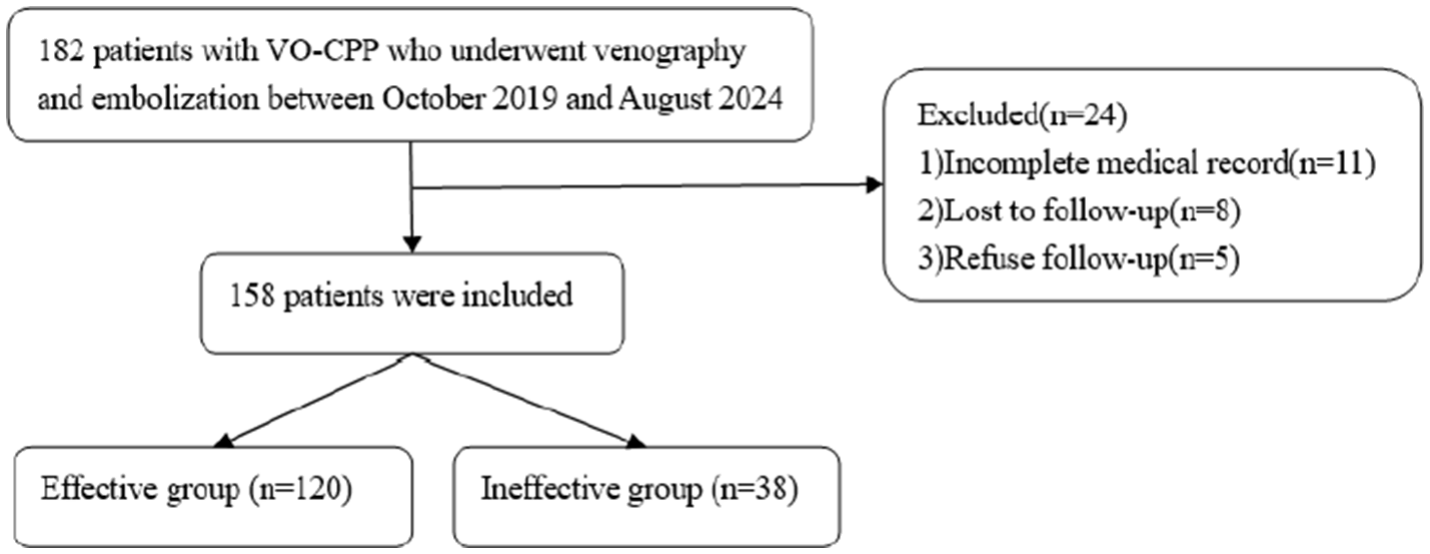
Study flowchart.

The Human Ethics Committee of our institute granted approval for the study. This research is a retrospective analysis conducted at a single center. Between October 2019 and August 2024, we accessed our institutional database to identify patients with VO-CPP who underwent selective retrograde venography and embolization. The inclusion criteria are as follows: (1) chronic pain lasting more than 6 months; (2) Age ≥18 years; (3) with complete clinical data and pre-treatment images (ultrasound and MRI or CT venography). Patients with incomplete medical records or those who could not complete the follow-up were excluded. Initially, 182 patients were screened for eligibility, of which 24 were excluded, resulting in a final evaluation *n* of 158 patients (Figure 1).

### Interventional procedures

Embolization was conducted by two experienced investigators in interventional radiology, each with over 6 years of expertise. The patient was positioned supine, with the head turned 45 degrees to the left to facilitate access to the right side of the neck. Following disinfection and administration of local anesthesia (2% lidocaine), the right internal jugular vein was punctured using the modified Seldinger technique, and a 5-French (Fr) vascular sheath was implanted. During the Valsalva maneuver, a 0.035 inch hydrophilic-coated guidewire and an angiographic catheter were advanced to the ovarian vein ostium, performing a retrograde venogram. Subsequently, a 2.6 Fr microcatheter system was introduced through the catheter into the ovarian veins and venous plexus. The primary veins examined included the renal veins, ovarian veins, internal and external iliac veins, and pelvic escape veins. In each case selective bilateral ovarian venography was performed by one of the authors. The decision to perform embolization was made based on the angiographic findings. The condition for patients to receive interventional therapy were: (1) an ovarian vein diameter greater than 6 mm; (2) periuterine vein moderate dilation and mild tortuosity; (3) contrast retention exceeding 20 s. The angiographic catheter was then inserted into the distal ovarian vein, where the appropriate coils were placed according to the diameter and shape of the vein. The microcatheter was advanced through the coil into the ovarian venous plexus, and 3% polidocanol foam was utilized to embolize the refluxing veins until a significant reduction in flow was observed. Finally, the catheter was repositioned more proximally, employing the sandwich technique to embolize the ovarian veins. For patients with pelvic escape veins, escape point embolization or foam sclerotherapy was performed. Temporary balloon occlusion of the internal iliac veins was executed when necessary to minimize the risk of ectopic embolization.

### Medical therapy

Post-procedurally, patients were administered Aescuven forte (horse chestnut seed extract) 300 mg per oral twice daily for 28 days, for improving blood circulation. If necessary, additional oral pain medication was prescribed.

### Outcome assessment

Patients were followed up at 1 month, 3 months, 6 months, and then annually after embolization procedure through outpatient clinic visits and phone calls. The endpoint for follow-up was ineffective, pain recurrence or last follow-up time, whichever happened the earliest. Pain intensity was assessed before and after treatment using the VAS, with total scores ranging from 0 (no pain at all) to 10 (worst imaginable pain). The spectrum of relative improvement was categorized into five distinct ranges: no improvement or worsening (≤0), minimal improvement (>1–25%), fair improvement (>25–50%), good improvement (>50–75%), and excellent improvement (>75–100%).

The primary outcome was the proportion of patients achieving an effective response, defined as a decrease in baseline VAS of at least 50% at the last follow-up. Secondary outcomes included technical success, time to onset of pain relief, complication rates, and risk factors for poor prognosis.

### Statistical design and analysis

The analysis of statistics was conducted using SPSS version 21.0. Normally distributed variables are presented as Mean ± Standard Deviation (SD) and skewed data as Median and Interquartile Range (IQR), while categorical variables are expressed as counts and percentages. The means of continuous variables were compared using *t*-tests or the Mann–Whitney *U* test when suitable. Categorical variables were analyzed with the Chi-square test or Fisher’s exact test. To identify clinically significant factors that may be linked to poor prognosis, binary logistic regression analysis was performed. A *p*-value of less than 0.05 was considered to indicate statistical significance.

## Results

### Clinical baseline data

The clinical baseline data are presented in Table 1. All patients included in the study were multiparous, the median number of children was 2, with a maximum of 7 children. They experienced dull pain of varying intensity, which was noncyclical and persisted for more than 6 months. The predominant symptoms reported were chronic pelvic pain (100%), lumbosacral pain (87.3%), congestive dysmenorrhea (42.7%, 53/124), dyspareunia (46.8%), and urinary frequency and urgency (60.1%), as well as perineal pain (78.5%). The baseline median VAS score was recorded at 7.0 (IQR 8-7).

**Table 1:** Clinical baseline data.

Parameter	Number (%)
Age	43.3 ± 11.0
Disease duration	
≥1 year	115 (72.8%)
<1 year	43 (27.2%)
Parity, median (IQR)	2.0 (3–2)
BMI	22.8 ± 4.1
≥24	51 (32.3%)
<24	107 (67.7%)
VAS score, median (IQR)	7.0 (8–7)
Symptoms	
Chronic pelvic pain	158 (100%)
Lumbosacral pain	138 (87.3%)
Congestive dysmenorrhea	53 (42.7%, 53/124)
Dyspareunia	74 (46.8%)
Urinary frequency and urgency	95 (60.1%)
Perineal pain	124 (78.5%)
Menopause	28 (17.7%)
Hysterectomy	6 (3.8%)
Lower limb varicose vein	15 (9.5%)
Estrogen-dependent disease	51 (32.2%)
Fibroids	31 (19.6%)
Adenomyosis	24 (15.2%)
Endometriosis	13 (8.2%)
Polycystic ovarian syndrome	8 (5.1%)

### Embolization

**Figure 2:**
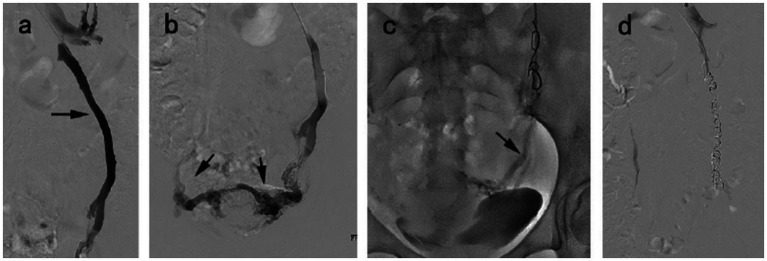
A 38 year-old woman diagnosed with VO-CPP and symptoms duration of 2 years. **(a)** Venography of the left ovarian vein showed venous distension, reflux (black arrow). **(b)** Congestion of the pelvic venous plexus. **(c)** The microcatheter (black arrow) was inserted into pelvic venous plexus via the previously placed coil to inject 3% polidocanol foam. **(d)** The disappearance of venous reflux following embolization.

**Figure 3:**
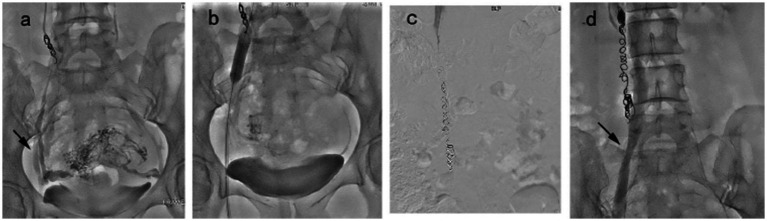
Embolization procedure in a 43 year-old patient was diagnosed with VO-CPP. **(a)** Congestion of the pelvic venous plexus and opacification of bilateral internal iliac vein (black arrow). **(b)** A balloon catheter was placed in the right internal iliac vein to prevent the return of the foam hardener until blood flow was reduced significantly. **(c)** Right ovarian vein embolization was performed using the sandwich technique. **(d)** The balloon catheter was withdrawn, and angiography showed no abnormality (black arrow).

**Figure 4:**
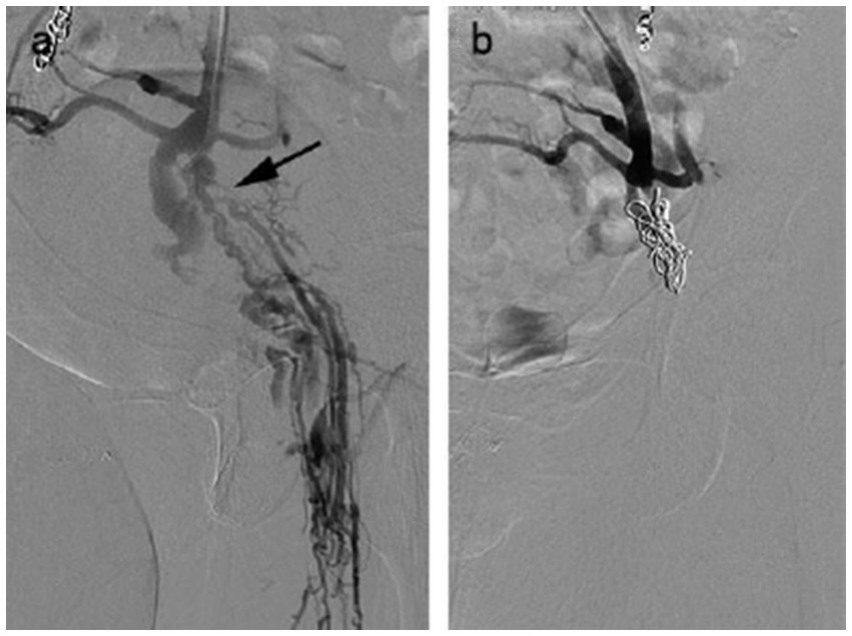
A 58 year-old female with venous escape points. **(a)** Pelvic escape veins (inguinal points) were confirmed by intraprocedural venography. **(b)** No abnormal blood flow was observed after escape point embolization.

Embolization technical success was 100%. One hundred fifty-eight patients were found to have reflux of single or multiple veins, and no cases of blood reflux were found under standardized Valsalva in the pelvic veins at post-embolization (Figure 2). The procedural details are shown in Table 2. Bilateral embolization of the ovarian veins was performed in 38.6% of all patients. For reducing ectopic embolization, temporary balloon occlusion of internal iliac veins was performed using a balloon catheter (Figure 3). Embolization of pelvic escape veins was performed in 4 patients (Figure 4). The mean procedural time, from local anesthesia to catheter removal, was 52 ± 25 min (range, 38–125 min).

**Table 2:** Procedure details.

Parameter	Number (%)
Left ovarian vein diameter	8.4 ± 2.3 mm
Right ovarian vein diameter	6.5 ± 1.8 mm
LOV only	66 (41.8%)
ROV only	31 (19.6%)
LOV and ROV	61 (38.6%)
Temporary internal iliac balloon occlusion	15 (9.5%)
Number coils (mode)	4
Escape points (number)	4 (2.5%)
X-ray fluoroscopy time (min)	13.6 ± 4.3
Total radiation dose (mGy)	325.4 ± 76.1
Total operation time (min)	52.6 ± 25.5

### Follow-up

In 141 cases (89.2%), clinical symptoms were optimized 2–3 months post-treatment, with significant improvement in patient symptoms lasting for 6 months.

The median VAS scores before treatment and at 1 month, 3 months, and 6 months post-treatment, as well as at the last follow-up (mean 15.7 ± 10.1 months, rang 6.0–42.0 months), were recorded as 7.0 (IQR8-7), 5.0 (IQR7-4), 4.0 (IQR6-3), 3.0 (IQR4-3), and 2.0 (IQR3-2), respectively. The VAS scores at each time point following embolization procedure were significantly lower than those recorded prior to embolization procedure, with all differences being statistically significant (*p* < 0.001). The proportions of clinical success at 6 months post-treatment and at the last follow-up were 83.5 and 75.9%, respectively. Notably, 90.9% of patients who experienced clinical success at 6 months continued to maintain their response at the last follow-up.

At the last follow-up, the distribution of improvement among patients was as follows: 15.8% (25 cases) exhibited excellent improvement, 60.1% (95 cases) demonstrated good improvement, 11.4% (18 cases) showed fair improvement, 1.9% (3 cases) indicated minimal improvement while 10.8% (17 cases) experienced no improvement (12 cases) or worsening (5 cases).

### Adverse events

There were 11 cases (7.0%) of embolization-related complications, including 2 cases of coil migration (which were successfully snared and recovered), 4 cases of parametrial/uterine veins thrombosis, 2 cases of localized hematoma, and 3 cases of mild contrast reactions. Importantly, no serious complications occurred during the intraoperative or postoperative periods.

### Clinically relevant risk factors for long-term prognosis

In the univariate analysis, multiparity (*p* = 0.007), perineal pain (*p* = 0.027) and estrogen-dependent disease (*p* = 0.002) were found to be associated with the clinical efficacy of embolization (see Table 3). The multivariate analysis indicated that parity ≥3 (OR, 2.266; 95% CI: 1.034–4.969; *p* = 0.041) and estrogen-dependent disease (OR, 2.586; 95% CI: 1.177–5.680; *p* = 0.018) were independent risk factor for poor prognosis.

**Table 3:** Univariate analysis of the prognostic factors related to patients with clinical efficacy.

Variables	Factors	Effective group (*n* = 120)	Ineffective group (*n* = 38)	*p*-value
Age (y)		43.8 ± 10.8	41.6 ± 11.8	0.276
Disease duration	≥1 year	90 (75.0%)	25 (65.8%)	0.266
	<1 year	30 (25.0%)	13 (34.2%)	
Parity	≥3	46 (38.3%)	24 (63.2%)	0.007
	<3	74 (61.7%)	14 (36.8%)	
BMI		22.9 ± 4.1	22.4 ± 4.1	0.589
Pelvic pressure	No	2 (1.7%)	0 (0%)	1
	Yes	118 (98.3%)	38 (100.0%)	
Lumbosacral pain	No	15 (12.5%)	4 (10.5%)	0.968
	Yes	105 (87.5%)	34 (89.5%)	
Dyspareunia	No	66 (55.0%)	18 (47.4%)	0.411
	Yes	54 (45.0%)	20 (52.6%)	
Bladder symptoms	No	48 (40.0%)	15 (39.5%)	0.954
	Yes	72 (60.0%)	23 (60.5%)	
Perineal pain	No	75 (62.5%)	16 (42.1%)	0.027
	Yes	45 (37.5%)	22 (57.9%)	
Embolization method	Left	52 (43.3%)	14 (36.8%)	0.778
	Right	23 (19.2%)	8 (21.1%)	
	Bilateral	45 (37.5%)	16 (42.1%)	
Left ovarian vein diameter		8.3 ± 2.3	8.4 ± 2.5	0.873
Right ovarian vein diameter		6.5 ± 1.9	6.6 ± 1.8	0.645
Estrogen-dependent disease	No	89 (74.2%)	18 (47.4%)	0.002
	Yes	31 (25.8%)	20 (52.6%)	

## Discussion

Our research confirms that coil and foam embolization is a feasible and safe endovascular procedure for the treatment of VO-CPP, providing relatively early symptom relief for most patients within 2-3 months. At the last follow-up, 75.9% of patients reported improvement or complete resolution of pain, with the median VAS score decreased from a median of 7.0 to a median of 2.0. While some patients respond positively to the embolization intervention, others may experience recurrences after the primary therapy, with pain-related symptoms recurring in 9.1% of patients within 6–15 months. Additionally, we found that parity ≥3 and estrogen-dependent disease was associated with a poorer prognosis.

Currently, the technical success rate of embolization for VO-CPP has reached 94–100% (14, 20). However, there is a notable lack of uniform standards for prognostic assessment. Among the symptoms associated with VO-CPP, chronic pelvic pain is the most frequently reported (20). While embolization coils may provide some symptom relief, the time to remission varies considerably, ranging from 1 day to 6 months. Liang et al. (9) reported that 77% of patients experienced pain-related symptom relief within 1 month, and 89% showed significant clinical improvement after 6 weeks. Laborda et al. (7) noted that the VAS scores of patients decreased the most within 6 months post-treatment, with 93% of patients demonstrating symptom improvement. Furthermore, previous studies (7, 8, 10, 17, 21, 22) have reported significant improvements in VAS scores for pelvic pain when comparing pre-procedural and post-procedural assessments (*p* < 0.05). Collectively, these studies indicate that embolization can lead to substantial symptom improvements for a large percentage of patients, with 67–100% of participants reporting a reduction in pelvic pain following treatment. A recent meta-analysis (14) also confirmed that pelvic venous embolization is a promising treatment modality for VO-CPP, either alone or in combination with other therapies, as all included studies demonstrated symptom improvement in the majority of patients, alongside low complication and recurrence rates. The results of our study align with these previous findings, demonstrating the effectiveness of selective embolization of refluxing veins, which resulted in high rates of symptom relief and improvement in quality of life. Additionally, we also avoided unnecessary embolization of antegrade non-refluxing pathways. Only embolize refluxing veins offers shorter procedure times, reduced radiation exposure, lower treatment costs, and a simplified technique in comparison to extensive embolization (bilateral ovarian and internal iliac veins).

The safety profile of combined therapy is generally favorable, with minor complications such as post-procedural pain, access site hematoma, and venous perforation reported, though these typically lack significant clinical repercussions (23). However, some studies have identified more serious complications, including coil migration and thrombosis of pelvic veins, which necessitate careful patient selection and monitoring following the procedure (7, 19, 24). Overall complication rates range from 0 to 9.0%, with most complications being manageable (14, 19). In our study, the complication rate for pelvic venous embolization was 7%, which aligns with findings from other studies. Notably, the most common major complication of combined therapy was thrombosis of pelvic veins rather than coil migration. This phenomenon may be attributed to venous thrombosis induced by coil embolization, which is exacerbated by reduced blood flow into the lesion following sclerotherapy (25, 26). Consequently, the use of anticoagulant drugs for a specified period post-treatment may represent a reasonable preventive strategy.

The efficacy of embolization is supported by long-term follow-up studies, which indicate that symptom relief can persist for several years post-treatment. These clinical responses were maintained after a follow-up of 5 years in two studies, with response rates of 93.9 and 84.4%, respectively (7, 8). This long-term efficacy is crucial for patients suffering from chronic pelvic pain, as it suggests that embolization not only provides immediate relief but also contributes to lasting improvements in their condition. In addition, we found that patients with multiparity exhibited poorer outcomes; recurrence following coil embolization is related to vein morphology and compromised hemodynamics. This may be attributed to the significant amounts of estrogen and progesterone produced by the corpus luteum and placenta during pregnancy, leading to pelvic vein dilation (with the volume of pelvic veins increasing by 60%). Under chronic stress, vessel morphology cannot revert to its original state, resulting in irregularly dilated and tortuous vessels that cause reverse blood flow, congestion, and stasis (4, 27, 28). Therefore, a logical approach to treating VO-CPP is to address the root cause of the congestion, specifically chronic venous reflux (9, 22). We assume that hemodynamics cannot be optimized in multiparous women through only embolize refluxing veins; extensive embolization may be necessary. In addition, we also found that venous embolization therapy is less effective for VO-CPP patients with hormone-dependent diseases such as uterine fibroids, endometriosis and adenomyosis, as these diseases have had overlapping, interactive, cumulative effects upon chronic pain. This highlights the importance of addressing comorbidities while dealing with venous diseases.

In addition to pain relief, combined therapy has been associated with improvements in other symptoms related to VO-CPP, such as vulvar swelling and lower limb edema (13, 29–31). The procedure effectively addresses the underlying venous insufficiency, which is often exacerbated by factors such as hormonal changes during pregnancy and the anatomical predisposition of pelvic veins (11, 13).

However, this study has some limitations. First, although 158 patients were enrolled, the follow-up period was not sufficiently long, the longer-term effects require further study. Second, the analgesics use was not included as an end-point in the research. Finally, a well-designed, multicenter, prospective, randomized study is needed to validate our findings.

In conclusion, embolization represents a significant advancement in the management of PeVD, offering a minimally invasive option with high rates of symptom relief and a favorable safety profile (15). As more data becomes available, particularly from larger and more diverse patient populations, the understanding of the long-term benefits and potential risks associated with this treatment will continue to evolve, further solidifying its role as a first-line therapy for VO-CPP.

## Data Availability

The original contributions presented in the study are included in the article/supplementary material, further inquiries can be directed to the corresponding author.
